# *Litomosoides brasiliensis* (Nematoda: Onchocercidae) infecting chiropterans in the Legal Amazon region, Brazil

**DOI:** 10.1590/S1984-29612022059

**Published:** 2022-11-28

**Authors:** Thaliane França Costa, Danielle Jordany Barros Coutinho, Ana Karoline Sousa Mendes Simas, Gabriella Vieira dos Santos, Rita de Maria Seabra Nogueira, Francisco Borges Costa, Maria Claudene Barros, Elmary da Costa Fraga, Andréa Pereira da Costa

**Affiliations:** 1 Laboratório de Parasitologia e Doenças Parasitárias dos Animais, Programa de Pós-graduação em Ciência Animal, Universidade Estadual do Maranhão – UEMA, São Luís, MA, Brasil; 2 Laboratório de Genética e Biologia Molecular (Genbimol), Programa de Pós-graduação em Ciência Animal, Universidade Estadual do Maranhão – UEMA, Caxias, MA, Brasil

**Keywords:** Nematoda, bats, filariids, Brazil, Nematoda, morcegos, filarídeos, Brasil

## Abstract

Chiropterans play an important role in the maintenance of the environmental balance, since they are pollinators, seed dispersers and predators. They contribute to transmission and spreading of microorganisms such as helminths, fungi, protozoa, bacteria and virus. The aim of the present study was to investigate natural filariid infection among bats in the Legal Amazon region, Brazil, by means of parasitological and molecular analyses. Blood samples were collected from 82 bats for blood smears and for DNA extraction via the polymerase chain reaction (PCR) assay. Microfilariae were observed in blood smears from *Carollia perspicillata* (2), *Artibeus lituratus* (1), *Artibeus fimbriatus* (2), *Dermanura gnoma* (2) and *Glossophaga soricina* (1). Five positive samples were detected through the PCR assay and four of these were also positive in blood smears. From genome sequencing and comparative analysis with sequences deposited in GenBank, one sample showed 99.31% similarity to the species *Litomosoides brasiliensis*. The present study expands the geographical distribution of *L. brasiliensis*, to include the state of Maranhão as an area of occurrence of this species and includes *D. gnoma* and *A. fimbriatus* as hosts in Brazil*.*

## Resumo

Os quirópteros desempenham um papel importante na manutenção do equilíbrio ambiental, pois são polinizadores, dispersores de sementes e predadores. Contribuem para a transmissão e disseminação de microrganismos, como helmintos, fungos, protozoários, bactérias e vírus. O objetivo do presente estudo foi investigar a infecção natural por filariídeos em morcegos na região da Amazônia Legal, Brasil, por meio de análises parasitológicas e moleculares. Amostras de sangue foram coletadas de 82 morcegos para confecção de esfregaços e para extração de DNA para a Reação em Cadeia da Polimerase (PCR). Microfilárias foram observadas em esfregaços sanguíneos de *Carollia perspicillata* (2), *Artibeus lituratus* (1), *Artibeus fimbriatus* (2), *Dermanura gnoma* (2) e *Glossophaga soricina* (1). Cinco amostras positivas foram detectadas por meio do ensaio de PCR, e quatro destas também foram positivas em esfregaços de sangue. A partir do sequenciamento do genoma e análise comparativa com sequências depositadas no GenBank, uma amostra apresentou 99,31% de similaridade com a espécie *Litomosoides brasiliensis*. O presente estudo expande a distribuição geográfica de *L. brasiliensis*, abrangendo o estado do Maranhão como área de ocorrência desta espécie, e inclui *D. gnoma* e *A. fimbriatus* como hospedeiros no Brasil.

## Introduction

Brazil has a diverse chiropteran fauna that includes 9 families, 68 genera and 179 species (Feijó et al., 2015; Nogueira et al., 2014; Paglia et al., 2012). These form an important component of the mammalian fauna of Neotropical ecosystems, with regard to their richness and species diversity (Kalko, 1998; Marinho-Filho & Gastal, 2001). Moreover, bats play an important role in maintaining the environmental balance, since they are pollinators, seed dispersers and predators (Bianconi et al., 2004; Kalko, 1998; Menezes et al., 2015; Pedrozo et al., 2016). They are also considered to be important reservoirs of emerging and re-emerging zoonotic pathogens. Therefore, continuous surveillance and monitoring of these animals is essential in order to prevent pandemics (Brook & Dobson, 2015; Kadam et al., 2021; León et al., 2021).

Bats’ characteristics of high flight mobility, great diversity of shelters, interaction among species in the same environment and remarkable social behavior make them interesting subjects for scientific studies. They contribute to transmission and spreading of microorganisms such as helminths, fungi, protozoa, bacteria and virus (Cabral et al., 2013; Frick et al., 2016; Roque & Jansen, 2014; Savani et al., 2009; Zetun et al., 2009).

Studies on parasite biodiversity are important for species management and conservation, since parasitism plays an essential role in ecosystems, through regulating host density, stabilizing food chains and structuring communities of animals (Poulin & Morand, 2000). Because of the high relevance of parasite diversity to ecosystems, any report on parasite infection provides understanding about ecology and environmental impacts (Rendón-Franco et al., 2019).

In Brazil, 59 species of helminths have been reported as infecting bats: 28 species of nematodes, 23 of trematodes, 6 of cestodes and 2 of acanthocephalans (Cardia et al., 2015; Santos & Gibson, 2015). The state of Maranhão is located in the Legal Amazon region, with a strategic position at the confluence of the Cerrado, Caatinga and Brazilian Amazon biomes, and comprises a mosaic of landscapes that are rich in fauna and flora. In this Neotropical region, studies have been conducted to provide data on chiropteran species (Bernard et al., 2011; Cruz et al., 2007; Olímpio et al., 2016). However, despite the high species diversity, studies on internal parasites in these biomes are scarce.

The aim of the present study was to investigate filariid species among chiropterans in the Legal Amazon region, in order to contribute to knowledge about the biodiversity of filariids among bats in Brazil.

## Materials and methods

### Study area

**Figure 1:**
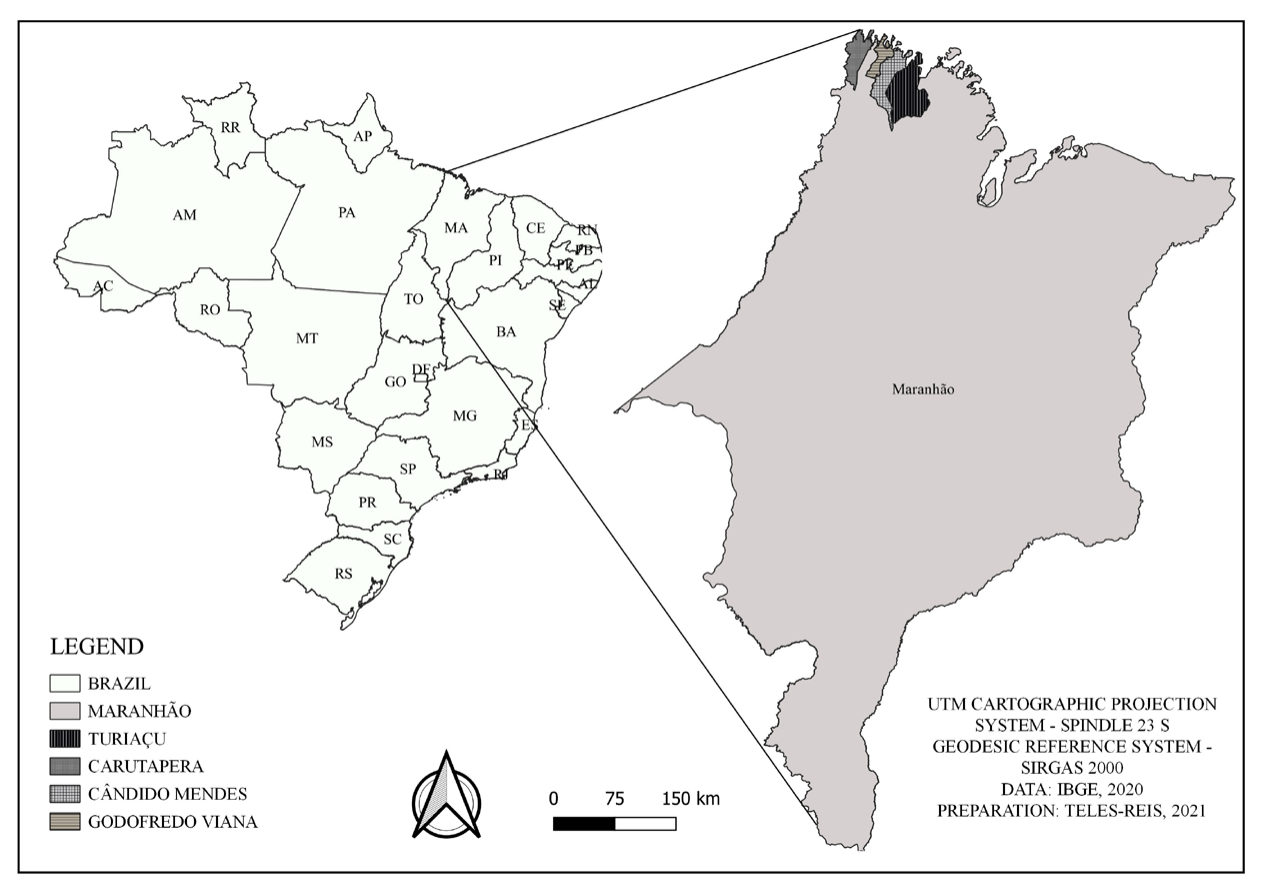
Municipalities of Turiaçu, Cândido Mendes, Godofredo Viana and Carutapera, in the Gurupi microregion of the state of Maranhão, northeastern Brazil.

This study was carried out in the municipalities of Turiaçu (1°39’44’’ S; 45°23’30’’ W), Cândido Mendes (1°26’34’’ S; 45°43’19’’ W), Godofredo Viana (1°24’19’’ S; 45°46’49’’ W) and Carutapera (1°12’11’’ S; 46°01’60’’ W), which are in the Gurupi microregion of the state of Maranhão, northeastern Brazil (Figure 1).

### Sample collection

Sampling periods were selected based on the lunar calendar and were concentrated in April 2017 in Godofredo Viana and Cândido Mendes and in March 2018 in Turiaçu and Carutapera.

Bats were collected between 18:00 and 00:00, over 5-day periods, with the aid of mist nets. They were then anesthetized with ketamine, at a dose according to their weight, photographed and identified. Specimen identification was performed based on specialized classification (Dos Reis et al., 2007; Miranda et al., 2011; Uieda, 2008). Approximately 2 mL of blood were collected from each specimen by means of cardiac puncture, for blood smears and molecular assays.

### Parasitological detection of filariids

Blood smears were made in triplicate, fixed with methanol, stained with Giemsa and analyzed under an optical microscope at 40x and 100x magnification. The blood smears were scanned to search for microfilariae.

### Molecular assay

Genomic DNA was extracted from blood, in accordance with the protocol established for use in the Genejet Genomic DNA purification kit (Thermo Scientific, UAB, Lithuania), with previous incubation of 0.5 g of tissue, 20 μL of proteinase enzyme K (20 mg/ml) and 700 μL of lysis solution. Fragments of the gene 12S rDNA were amplified by means of the polymerase chain reaction (PCR), using the primer pairs Fila12SF (5’-CGGGAGTAAAGTTTTGTTTAAACCG-3’) and Fila12SR (5’-CATTGACGGATGGTTTGTACCAC-3’), as described by Otranto et al. (2011), which amplify fragments of 330 bp from preserved filariids.

The amplified products were subjected to horizontal electrophoresis at 50 V/100 mA in Tris-acetate-EDTA (TAE) buffer, on 1.5% agarose gel stained with SYBR Safe (Invitrogen). The bands were viewed and photographed using a UV light transilluminator. The amplified products were purified using the ExoSAP-IT commercial product (USB Corporation), consisting of Exonuclease I (Exo I) to digest excess primers and shrimp alkaline phosphatase (SAP) to degrade excess nucleotides from the PCR. After purification, sequencing was performed using the Big Dye Terminator kit (Perkin Elmer), in accordance with the manufacturer’s specifications, in an ABI PRISM 3500 automated sequencer (Life Technologies). The sequences obtained were edited in the SeqMan software (Lasergene, DNAstar, Madison, Wisconsin, United States) and were subjected to similarity analysis using the Basic Local Alignment Search Tool (BLAST two-sequence analysis) to verify their homology with corresponding sequences available in GenBank (Altschul et al., 1990).

## Results

Among the 82 bat specimens that were caught, 24 (29.26%) were obtained from the municipality of Godofredo Viana (23 from the family Phyllostomidae and one from Vespertilionidae). In Turiaçu, 14 (17.07%) were caught, all of them from the family Phyllostomidae; 26 (31.70%) from Candido Mendes (25 Molossidae and 1 Phyllostomidae); and 18 (21.95%) from Carutapera (all of them in the family Phyllostomidae).

In this study the following genera/species were identified: *Artibeus fimbriatus*, *Dermanura gnoma*, *Uroderma bilobatum*, *Glossophaga soricina*, *Molossus molossus*, *Molossus* sp., *Artibeus* sp., *Carollia* sp., *Dermanura* sp. and *Myotis nigricans*. Among the bats recorded in all areas, the most frequent were *Artibeus* sp. (26/82) and *Molossus molossus* (24/82) (Table 1).

**Table 1:** Distribution of bat genera/species captured in the Municipalities of Godofredo Viana, Turiaçu, Cândido Mendes e Carutapera, Maranhão, Brazil.

GENERA/SPECIES	FAMILY	GODOFREDO VIANA	TURIAÇU	CÂNDIDO MENDES	CARUTAPERA	TOTAL
*Artibeus fimbriatus*	Phyllostomidae	-	01	-	-	01
*Artibeus* sp.	Phyllostomidae	11	03	01	11	26
*Carollia* sp.	Phyllostomidae	08	-	-	02	10
*Dermanura gnoma*	Phyllostomidae	-	02	-	01	03
*Dermanura* sp.	Phyllostomidae	01	03	-		04
*Glossophaga soricina*	Phyllostomidae	03		-	04	07
*Molossus molossus*	Molossidae	-	-	24	-	24
*Molossus* sp.	Molossidae	-	-	01	-	01
*Myotis nigricans*	Vespertilionidae	01	-	-	-	01
*Uroderma bilobatum*	Phyllostomidae	-	05	-	-	05
TOTAL	-	24	14	26	18	82

**Figure 2:**
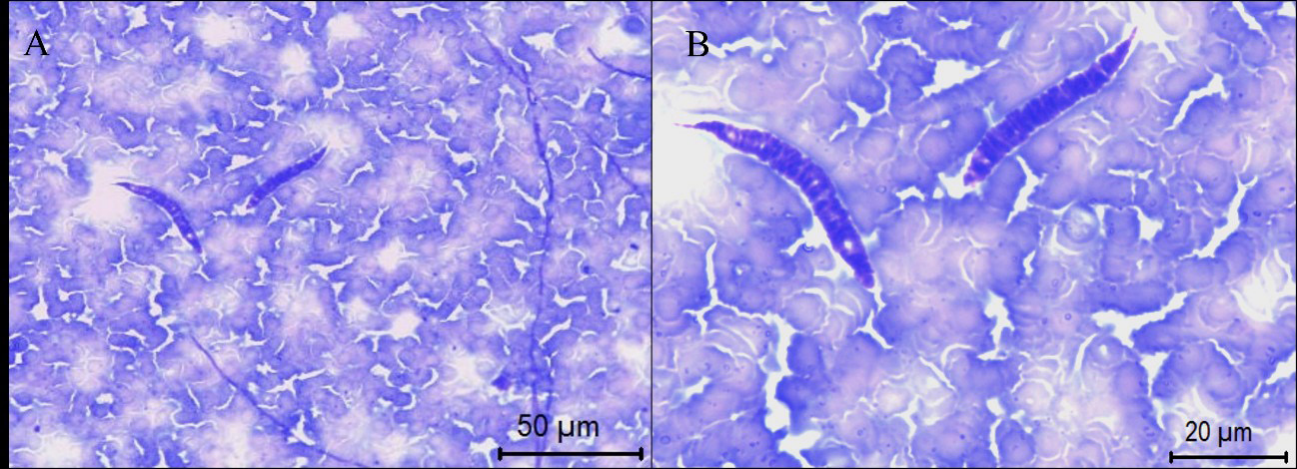
Microfilariae in blood smears from bats, state of Maranhão, northeastern Brazil. (A) [x400] and (B) [x1000].

Microfilariae (Figure 2) were observed in blood smears from bats of the species *Carollia perspicillata* (2 specimens), *Artibeus lituratus* (1), *Artibeus fimbriatus* (2), *Dermanura gnoma* (2) and *Glossophaga soricina* (1). Except for *G. soricina*, which is a nectar-feeding bat, the other species are fruit bats.

From the PCR assays, five positive samples were detected; four of these were also found to be positive through blood smears. From genome sequencing and comparative analysis with sequences deposited in GenBank, one was found to show 99.31% similarity to the species *Litomosoides brasiliensis* (accession number: MW471081.1).

## Discussion

The family Phyllostomidae was the most representative (68%) among the bats sampled, followed by Molossidae (30.66%). The high frequency of catching specimens of Phyllostomidae was due to its diversity in Brazil: this family accounts for approximately 57% of all bat species in this country.

According to Anderson & Bain (2009), the family Onchocercidae is composed of seven subfamilies, in which five genera have already been identified in bats: *Litomosoides*, *Migonella*, *Chiropterofilaria*, *Josefilaria* and *Litomosa* (Bain et al., 2002). The genus *Litomosoides*, which includes at least 31 species (Guerrero et al., 2002), and the genus *Migonella* are found in bats in South America (Santos & Gibson, 2015).

In studies conducted in Brazil, parasitism by species of *Litomosoides* in bats of the genus *Artibeus* and *Carollia* has also been reported (Albuquerque et al., 2015; Cardia 2012; Costa et al., 2021; Mello, 2017; Vicente et al., 1997). However, these studies consisted of reports on adult parasites and not microfilaria in blood samples. In the present study, the diagnosis was based on findings of microfilaria in blood smears and on PCR-positive samples and genome sequencing.

Presence of microfilaria in peripheral blood allows transmission of the parasite within the bat population, both though hematophagous arthropods and transplacentally (Gazarini et al., 2012; Williams, 1948). Transmission is facilitated through the bats’ habits, since they live in groups and use caverns and trees as shelters (Melaun et al., 2014; Sekiama et al., 2013).

Although the vectors of bat filariids are not well known, certain species of *Litomosoides* are transmitted by hematophagous mites (Williams, 1948), such as those in the families Dermanyssidae (Anderson, 2000) and Macronyssidae (Bain et al., 2002; Guerrero et al., 2002). The latter family is also considered to act as vectors for filariids in rodents and marsupials (Guerrero et al., 2002).

*Litomosoides* spp. have also been detected through PCR on blood samples from *Artibeus jamaicensis* and through PCR on genetic material from *Trichobius intermedius* (Diptera: Streblidae) and *Periglischrus iheringi* (Acari: Spinturnicidae). However, it was not possible to incriminate these as vectors (Reeves et al., 2016).

Adults of the genus *Litomosoides* include species that parasitize the thoracic and abdominal cavities of phyllostomid and mormoopid bats; cricetid, sciurid and hystricognath rodents; and didelphid marsupials (Brant & Gardner, 2000; Forrester & Kinsella, 1973; Guerrero et al., 2002; Notarnicola & Navone, 2011).

In the present study, microfilariae of *Litomosoides* sp. were found in blood smears from *C. perspicillata*, *A. lituratus*, *A. fimbriatus*, *D. gnoma* and *G. soricine*. Reports of *Litomosoides* sp. in association with the genera *Artibeus* and *Carollia* are not uncommon; in fact, among chiropterans, this nematode seems to prefer host of the genus *Artibeus* (Guerrero et al., 2002; Jiménez et al., 2017). These parasites are limited to only a few host species and are order specific. Thus, species that occur in bats do not occur in rodents and marsupials, and vice versa (Brant & Gardner, 2000; Guerrero et al., 2002; Vogeler et al., 2018).

It is important to highlight that among the bat species that presented microfilariae, four were fruit bats and one was a nectar-feeding bat. Both of these types of bat are fundamental for the biosystem and ecosystem, since they fly long distances searching for fruits and they disperse seeds along the way and act as pollinators. Hence, they are essential for forest regeneration and maintenance of plant diversity and ecosystem equilibrium (Estrada & Coates-Estrada, 2001; Medellin & Gaona, 1999). However, when infected by *Litomosoides*, they can present signs of weakness, tachypnea and hemorrhage, which leads to decreased efficiency of seed dispersion and pollination (Rendón-Franco et al., 2019).

The molecular analysis on the blood samples showed there was 99.31% similarity to *L. brasiliensis*. This species has been reported parasitizing the families Phyllostomidae and Vespertilionidae, which are widely distributed in South and Central (Albuquerque et al., 2015; Brant & Gardner, 2000; Costa et al., 2021; Guerrero et al., 2002; Jiménez et al., 2021; Mourão et al., 2002; Vicente et al., 1997; Vogeler et al., 2018). In Brazil, presence of *L. brasiliensis* has been reported in the states of Amapá, Minas Gerais, Mato Grosso, Mato Grosso do Sul, Pará, Piauí, Paraná, Rio de Janeiro and São Paulo (Albuquerque et al., 2015; Costa et al., 2021; Mourão et al., 2002). Now, we register it in the state of Maranhão. This study provides the first report of *L. brasiliensis* parasitizing *D. gnoma* and *A. fimbriatus* in Brazil.

Phylogenetic analyses have been used to reconstruct the evolution of *Litomosoides* and infer its history. Two decades ago, Brant & Gardner (2000) suggested that this genus was not a monophyletic group, although they identified 22 morphological characteristics with high levels of homoplasy. However, a recent study on a database of DNA sequences demonstrated that *Litomosoides* showed up as a monophyletic group that originated in Neotropical phyllostomid bats, with strong evidence of at least two events of host changing: one of them involving cricetid rodents and the other, mormoopids. The latter event included simultaneous geographical expansion of the parasite’s range (Jiménez et al., 2021). Thus, studies on molecular detection of *Litomosoides* are relevant, to provide data for reconstructing the phylogeny of supposed changes to hosts and monophyly.

The present study expands the geographical distribution of *L. brasiliensis*, to include the state of Maranhão as an area of occurrence of this specie. This study also includes *D. gnoma* and *A. fimbriatus* as hosts in Brazil*.*
